# Effects of Pasteurization and High-Pressure Processing of Camel and Bovine Cheese Quality, and Proteolysis Contribution to Camel Cheese Softness

**DOI:** 10.3389/fnut.2021.642846

**Published:** 2021-06-17

**Authors:** Mustapha Mbye, Huda Mohamed, Tholkappiyan Ramachandran, Fathalla Hamed, Ahlam AlHammadi, Rabih Kamleh, Afaf Kamal-Eldin

**Affiliations:** ^1^Department of Food Science, United Arab Emirates University, Al-Ain, United Arab Emirates; ^2^Department of Physics, United Arab Emirates University, Al-Ain, United Arab Emirates; ^3^Agthia Public Joint Stock Company (PJSC) Group of Companies, Al Ain, United Arab Emirates

**Keywords:** camel milk, bovine milk, cheese quality, pasteurization, high pressure processing

## Abstract

The effects of high-pressure processing (HPP) compared to thermal treatments on the quality of camel vs. bovine cheeses were studied. The study showed that camel milk has a lower microbial load compared to bovine milk, which is maintained during 7 days' storage of the processed milk. The effect of three HPP treatments (350, 450, and 550 MPa for 5 min at 4°C) and two pasteurization treatments (65°C for 30 min and 75°C for 30 s) on the quality of soft unripened camel and bovine milk cheeses were accessed. The cheeses were evaluated for pH, yield, proximate composition, textural and rheological properties, microstructure, and protein profile by SDS-PAGE electrophoresis. The effects of the treatments on cheese's hardness were different between the camel and bovine cheeses; while heat treatment at 65°C for 30 min gave the hardest bovine milk cheese (1,253 ± 20), HPP treatment at 350 MPa for 5 min gave the highest value for camel milk cheese (519 ± 5) (*p* < 0.05). The hardness of the cheeses was associated with low yield and moisture content. SDS-PAGE electrophoresis revealed that extensive proteolysis might have contributed to the softness of camel cheeses compared to bovine and suggested the involvement of some residual enzyme activities.

## Introduction

Nowadays, great attention is given to camel milk production and consumption because of its high nutritional value and digestibility (1). Bacterial fermentation and cheese manufacture are typical of perverse dairy products (2). Still, up to now, their application to camel milk is limited due to the extreme softness of the produced coagulum (3–5). The most crucial step in cheese making is the chymosin-induced coagulation of milk (6). The coagulation rate and the outcome of the cheese are significantly influenced by different factors, including the animal species and breed, the composition of the milk, and pretreatment of the milk such as pasteurization, homogenization, and pressure treatment (7). Milk pasteurization is an important step in cheese making to ensure the safety of the cheese (8). However, higher temperatures may lead to adverse effects on curd formation due to longer coagulation times and weaker gels (9, 10) and are less suitable for cheese production (7). Thus, non-thermal technologies, such as high-pressure processing (HPP), have emerged as alternatives to traditional heat treatment in milk and dairy products (11).

HPP provides a useful food preservation method that eliminates food bacteria by disrupting their cell membranes and the intermediate layer between the cell wall and the cytoplasmic membrane, deactivating membrane ATPase, and destroying the nucleic acids and ribosomes involved in protein synthesis (12). Unlike heat treatments, HHP also maintains the quality of fresh foods with little effect on flavor and nutritional factors such as vitamins and other bioactive compounds (13). HHP of milk induces electrostatic interactions between proteins leading to their disruption, solubilization of colloidal calcium phosphate, reduction in the size of casein micelles, and the whey protein's denaturation (14, 15). This modification was reported to improve the milk coagulation time and gel firmness of bovine milk cheese (15, 16). To the best of our knowledge, no data is available to describe the effect of HPP on camel milk's microbial load and milks utilization in cheese manufacture.

To explore a wider range of pressure-time combinations at a fixed temperature of 4°C, two experiments were performed. In the first experiment, the effect of pressure (350, 450, and 550 MPa) and time (3, 6, and 9 min) on microbial count load, cheese's yield, hardness, and viscosity were assessed. In the second experiment, the effects of the two pasteurization temperatures (65°C for 30 min, 75°C for 30 s) and three high-pressure treatments (350, 450, and 550 MPa for 5 min at 4°C) on the textural and physicochemical properties of cheeses made from camel and bovine milk were studied. Analysis of the cheeses by SDS-PAGE electrophoresis showed that proteolytic activities generate a large number of peptides in the camel but not bovine cheeses, which might be responsible for the softness of the camel milk cheeses.

## Materials and Methods

### Materials

Tank pooled raw camel and bovine milk samples were purchased from the Al Ain Dairy farm (Abu Dhabi Emirate, UAE). The milk was delivered to the Food Science Department at United Arab Emirates University in refrigerated coolers (4°C). The lyophilized yogurt starter culture used was Yoflex Express® 1.0, a 1:1 mixture of *Streptococcus thermophiles* and *Lactobacillus bulgaricus* subsp. *delbrückii*. Recombinant camel chymosin (CHY-MAX®M, activity of 1,000 IMCU/mL) was from Chr. Hansen (Hoersholm, Denmark). TEMED Ultra for molecular biology (N, N, N′, N′-Tetramethylethylenediamine, >99%), calcium chloride, and all other chemicals and reagents were of analytical grade and were purchased from Sigma-Aldrich (St. Louis, Missouri, USA). Unless otherwise stated, all the media and supplements used throughout microbial analysis are purchased from Oxoid (Oxoid, Basingstoke, Hampshire, England). Precision Plus Protein–unstained standard (molecular weight marker), 4 × Laemmli sample buffer (62.5 mM Tris-HCl, pH 6.8, 10% glycerol, 1% lithium dodecyl sulfate, 0.005% bromophenol blue), resolving gel buffer (1.5M Tris HCL, pH 8.8), stacking gel buffer (0.5 M Tris HCL, pH 6), sodium dodecyl sulfate (SDS) solution (10%), dithiothreitol (DTT), ammonium persulphate (APS), 10 × TGS buffer (0.25M Tris, 1.92M glycine and 1% sodium dodecyl sulfate), QC colloidal Coomassie stain and 30% acrylamide/Bis solution 29:1 (v/v) were purchased from Bio-Rad Laboratories Inc. (Hercules, CA, USA).

### Heat Treatments and High-Pressure Processing of Milk

The first experiment was performed using a central composite rotatable design with varying combinations of the independent variable pressure (308, 350, 450, 550, and 590 MPa at 4°C) and time (1, 3, 6, 9, and 10 mins) (17) and the response variables (cheese yield, hardness, and viscosity) were measured Table 1. Similarly, microbiological analyzes were carried out on the milk samples after various pressure-time combinations or after the pasteurization treatments **Table 5**. In the second experiment, the milk samples were subjected to two pasteurization regimes and three high-pressure treatment levels, as explained in Table 2. In this experiment, several other parameters were measured in addition to yield, hardness, and viscosity.

**Table 1:** Experimental design of the independent variables (pressure, time, and 4°C) and results of associated response variables (cheese yield hardness and viscosity).

**Run order**	**Independent variables**		**Response variables**					
	**Pressure** **(MPa)**	**Time** **(min)**	**Yield (g/100 g milk)**		**Hardness (g)**		**Viscosity (Pa.s)**	
			**Camel** **cheese**	**Bovine** **cheese**	**Camel** **cheese**	**Bovine** **cheese**	**Camel** **cheese**	**Bovine** **cheese**
1	550	3	15.8	19.2	271	684	7,990	10,358
2	590	6	16.3	20.6	212	583	7,819	9,499
3	450	6	13.4	16.0	334	810	8,778	13,488
4	450	1	12.9	15.8	362	843	8,821	13,188
5	450	6	13.6	16.3	341	812	8,766	13,452
6	450	6	13.4	16.8	343	819	8,758	13,423
7	450	10	14.0	18.8	311	783	8,655	13,288
8	350	9	12.0	15.2	519	927	9,431	16,699
9	350	3	12.4	15.5	498	900	9,212	16,241
10	450	6	13.5	17.1	337	814	8,722	13,417
11	308	6	12.2	15.4	507	913	9,330	16,441
12	550	9	16.0	19.8	243	652	7,919	10,058
13	450	6	13.7	16.6	335	822	8,768	13,488

**Table 2:** Chemical composition of camel and bovine milk cheeses prepared from pasteurized and HPP-treated milk^*^.

**Quality parameter**	**Camel milk cheeses**					**Bovine milk cheeses**				
	**LTLT, 65^**°**^C** **(30 min)**	**HTST, 75^**°**^C** **(30 s)**	**350 MPa** **(5 min at 4^**°**^C)**	**450 MPa** **(5 min at 4^**°**^C)**	**550 MPa** **(5 min at 4^**°**^C)**	**LTLT, 65^**°**^C** **(30 min)**	**HTST,** **75^**°**^C (30 s)**	**350 MPa** **(5 min at 4^**°**^C)**	**450 MPa** **(5 min at 4^**°**^C)**	**550 MPa** **(5 time at 4^**°**^C)**
Yield (%)	12 ± 0.02^g^	17 ± 0.3^c^	11.5 ± 0.2^h^	13.5 ± 0.2^f^	14.7 ± 0.3^e^	14 ± 0.3^e^	21 ± 0.2^a^	15 ± 0.15^d^	17 ± 0.4^c^	19 ± 0.31^b^
pH	5.3 ± 0.01^f^	5.2 ± 0.03^f^	5.5 ± 0.02^e^	5.6 ± 0.01^d^	5.8 ± 0.03^cd^	5.6 ± 0.04^d^	5.4 ± 0.02^e^	5.8 ± 0.02^bc^	5.9 ± 0.05^b^	6.4 ± 0.15^a^
Acidity (%)	2.7 ± 0.04^b^	2.9 ± 0.05^a^	2.5 ± 0.05^c^	1.9 ± 0.02^d^	1.7 ± 0.04^e^	1.2 ± 0.03^f^	1.3 ± 0.02^f^	1.1 ± 0.05^g^	0.8 ± 0.04^h^	0.7 ± 0.05^i^
Total solids (%)	40.9 ± 0.2^f^	37.4 ± 0.16^h^	40.6 ± 0.26^f^	39.7 ± 0.22^g^	39.4 ± 0.13^g^	53 ± 0.13^a^	41 ± 0.13^e^	51 ± 0.11^b^	49 ± 0.21^c^	47 ± 0.12^d^
Fat (%)	20.5 ± 0.1^g^	17.6 ± 0.37^i^	21.2 ± 0.1^f^	21.8 ± 0.13^e^	22.2 ± 0.12^e^	29 ± 0.09^a^	20 ± 0.08^h^	28 ± 0.05^b^	25 ± 0.1	23 ± 0.05^d^
Protein (%)	15.14 ± 0.1^de^	17.8 ± 0.03^c^	14.3 ± 0.3^e^	13.2 ± 0.41^f^	13.3 ± 0.21^f^	19.2 ± 0.3^a^	15.5 ± 0.31^d^	18.6 ± 0.24^abc^	18 ± 0.74^bc^	18 ± 0.45^ab^
Hardness (g)	367 ± 6^g^	228 ± 7^i^	519 ± 5^d^	341 ± 5^g^	276 ± 7^h^	1, 253 ± 20^a^	438 ± 14^e^	913 ± 9^b^	810 ± 12^c^	645 ± 7^d^
Cohesiveness	0.62 ± 0.02^c^	0.82 ± 0.02^a^	0.53 ± 0.01^d^	0.65 ± 0.01^bc^	0.68 ± 0.01^b^	0.37 ± 0.01^g^	0.52 ± 0.02^d^	0.41 ± 0.02^f^	0.45 ± 0.01^ef^	0.47 ± 0.02^e^
Gumminess (g)	228 ± 8^d^	185 ± 8^e^	281 ± 5^c^	224 ± 5^d^	187 ± 7^e^	463 ± 17^a^	227 ± 5^d^	374 ± 7^b^	356 ± 7^b^	305 ± 12^c^
Chewiness (MJ)	8.9. ± 0.36^ef^	3.7.8 ± 0.13^f^	12 ± 0.2^e^	7.7 ± 0.31^ef^	7.4 ± 0.21^ef^	99 ± 2.5^a^	51 ± 1.5^d^	93 ± 1.5^a^	79 ± 6.7^b^	59 ± 1^c^
Viscosity (Pa.s)	9, 717 ± 15^e^	3, 448 ± 235^i^	9, 804 ± 81^e^	8, 746 ± 22^f^	7, 945 ± 38^g^	17, 419 ± 119^a^	6, 859 ± 43^h^	16, 429 ± 240^b^	13, 539 ± 320^c^	12, 376 ± 327^d^
G-prime (pa.s)	58, 240 ± 150^e^	22, 657 ± 213^i^	58, 671 ± 252^e^	53, 429 ± 129^f^	46, 334 ± 350^g^	99, 360 ± 233^a^	38, 499 ± 186^h^	97, 753 ± 336^b^	81, 586 ± 351^c^	80, 201 ± 156^d^
G-double prime (pa.s)	22, 172 ± 129^e^	11, 336 ± 267^h^	23, 289 ± 139^d^	21, 424 ± 260^f^	20, 621 ± 308^g^	37, 694 ± 204^a^	11, 786 ± 153^h^	32, 173 ± 141^b^	24, 520 ± 164^c^	23, 353 ± 228^d^

* Values within a raw having different superscripts are significantly different (p < 0.05, n = 3 per treatment).

Values superscripts are significantly different (p < 0.05) with a the highest and i the lowest.

Heat treatments of milk samples were performed by low-temperature long time (LTLT, 65°C for 30 min) or high-temperature short-time (HTST, 75°C for 30 s) pasteurization. For the high-pressure processing (HPP), the two kinds of milk (camel and bovine) were filled in plastic bottles (330 mL) without any headspace and subsequently *vacuum* seal packed in polyethylene bags using a vacuum packaging machine Multivac Sepp C350 (Haggenmuller SE and CO. KG, Düsseldorf, Germany) before pressurizations. HPP treatments were performed using an Iso-Lab high-pressure pilot food processor S-FL-100-250-09-W (Stansted fluid power LTD Essex, UK). The HP unit consisted of a system that generates a maximum pressure of 700 MPa, an inlet and outlet temperature of 2–4°C, a pressure rate of 5 MPa/s, and a heating rate of (0.5°C/100 MPa). In this study, HPP was performed at three pressures (350, 450, and 550 MPa) at 4°C for different times as explained in experiments 1 and 2. The system was equipped with a water jacket that allows temperature control in the pressure chamber by circulating cold water. The pressure chamber was filled with distilled water as the transmitting fluid. The plastic bottles containing the milk samples were submerged in the pressure chamber and then subjected to varying combinations of pressure and time, as described in Tables 1, 2.

### Microbiological and Raw Milk Composition Analysis

Milk samples (25 ml) were diluted in buffered peptone saline (225 ml, 0.5% w/v; peptone; 0.85% w/v; NaCl), mixed in stomacher bag (Seward 400, England) for 2 min. To quantify the various microbial groups, Increased sensitivity to <1 CFU (colony-forming unit) per mL was achieved by spread plating 1 mL of the undiluted sample onto the agar media as well as the 1:10 dilutions to eliminate any inhibitory effect that may be present in the undiluted sample. Total plate count (TPC) was carried out on plate count agar (PCA), incubated at 32°C for 72 h (18). The coliforms were determined by the most probable number (MPN) method according to the US standard method (19). *Staphylococcus aureus* was enumerated on Baird Parker agar supplemented with egg yolk according to (20). *Listeria monocytogenes* were detected according to (21) while the *Escherichia coli* was examined with MacConkey agar followed by 24 h incubation at 37°C according to (22).

Lactose, protein, fats, and total solids contents (%) were evaluated using Near Infra-Red Multipurpose Analyzer (MPA), Bruker Optik Gmbh (Ettlingen, Germany) (23). The pH of the samples was determined using a digital pH meter (Starter 3100; Ohaus, New Jersey, USA), and the titratable acidity was determined in triplicate using the standard method ISO/TS 11,869:2,012 (IDF/RM 150:2,012) (3).

### Preparation of the Cheeses

Two liters of treated camel or bovine milk was processed into cheeses, three repetitions per treatment, supplemented with calcium chloride (3%) and incubated with 3% (w/v) of an active thermophilic yogurt starter culture at 43°C for 60 min to allow the pH to fall to 6.2 (3). Thereafter, recombinant camel chymosin (CHY-MAX®M, 50 IMCU) was added to the milk (24), and the incubation was continued for 4 h until the pH reached 4.8, and firm curd was observed. Then, the curd was placed in cheesecloth to drain for 8 h (25).

### Cheese Yield and Physicochemical Properties

The cheese yield was calculated as the percentage of weight recovered from the whole milk used for preparation (Yield = kg of fresh cheese ×100/mL of processed milk) (26). The pH of the samples was determined using a digital pH meter (OHAUS, Starter 3100, New Jersey, USA), and the titratable acidity was determined in triplicate using the standard method ISO/TS 11,869:2,012 [IDF/RM 150:2,012 (3)]. The texture profile analysis (TPA) of the cheese samples was analyzed using a CT III texture analyzer equipped with a 4.5 kg load cell (Brookfield, Middleborough, Massachusetts, USA). TPA was carried out with a compression test of the cheese in a 40 mL cup using a 25-mm-diameter perplex cylindrical probe (TA11/1000) with a test speed of 1 mm/s and 3 mm of target distance (3). The hardness (the amount of force required to attain a given deformation), cohesiveness (a measure of the extent to which cheese can be deformed before it ruptures), gumminess (the ability of cheese to regain its original position during the first deformation test), and chewiness (a measure of the energy required to masticate cheese into a uniform state before swallowing) were performed on cheese samples at room temperature (27).

The rheological properties measurement was carried out in a stress-controlled rheometer (Discovery Hybrid Rheometer, TA Instruments, Delaware, USA) fitted with cone plate geometry (30 mm diameter and 2° of inclination angle). Samples were loaded and spread on the horizontal plate's surface, and leftover pieces were trimmed off. The cheese was rested for 5 min to allow it to attain thermal equilibrium and stress relaxation. The top plate was slowly lowered until the gap was 1 mm. Strain sweep tests were conducted from 0.01 to 100% at a frequency of 1 Hz (3). The data obtained were elastic modulus (G′), viscous modulus (G″), and viscosity (Pa.s), which gave the viscoelastic range. Each measurement was performed in triplicate at a controlled temperature of 25°C using a water-cooling system (Thermo Cube Model 10–300-1CL, New York USA).

The microstructures of different cheese samples were observed using a JEOL JSM-6010LA scanning electron microscope (SEM, Akishima, and Tokyo, Japan). The lyophilized cheese samples were placed on an aluminum SEM stub with double-sided adhesive carbon tape and coated with gold. The samples were observed under a high vacuum and a voltage of 20 kV and recorded the micrographs of the pieces at a 400 × magnification (3).

### The Chemical Composition of the Cheeses and Wheys

The total solid, fat, and protein in camel and bovine milk cheese and whey samples were determined by near infra-red multipurpose analyzer using the equipment calibration model (MPA, Bruker Optik Gmbh, Ettlingen, Germany). All the samples were analyzed on the same day in triplicate. Fourier transform infrared (FTIR) spectroscopy of the cheese sample was analyzed with ATR-FTIR mid-infrared spectrometer (Nicole-TM 1S50 FTIR, Thermo Fisher Scientific, Massachusetts, USA). Three infrared spectra per sample were recorded between 4,000 and 400 cm^−1^ at a resolution of 4 cm^−1^. The dried cheese was ground and mixed with potassium bromide at 1:5 (sample: KBr). The pellet was then prepared, compressed, and scanned (28). The data was processed by OPUS/IR spectroscopic software installed on the FTIR system.

Protein analysis of camel and bovine cheese samples was performed using sodium dodecyl sulfate-polyacrylamide gel electrophoresis (SDS-PAGE) (29). Cheese samples were prepared using the method described before (30). Cheese samples (0.6 g) were dissolved in 25 ml. of 8M urea. The cheese samples were homogenized for 2 min using T 25 digital Ultra-Turrax (IKA-Werke GmbH and Co., KG, Staufen, Germany). To dissociate caseins, the urea-cheese dispersion was incubated in a temperature-controlled water bath at 37°C for 2 h and then defatted by centrifugation at 9,150 g at 4°C for 35 min and filtered through Whatman no. 1 filter paper (pore size, 11 μm). Of the filtered sample, a 10 μl portion was added to 30 μl of 4X Lamelli buffer solution containing 50 mM Dithiothreitol (added freshly). The sample and sample buffer mix were heated in a temperature-controlled water bath for 5 min at 90°C. From this mix, 6 μl was loaded on the hand-cast polyacrylamide gels.

Gels with 1 mm thickness were prepared using the gel hand casting accessories provided with the Bio-Rad Mini- PROTEAN Tetra cell (Bio-Rad Laboratories Inc., Hercules, California, USA). A 12% resolving gel and 4% stacking gel were prepared. To prepare a quantity of 15 ml of 12% resolving gel solution the following were added: 6 ml 30% acrylamide/Bis Solution 29:1, 3.75 ml 1.5M Tris HCL (pH 8.8), 150 μl 10% SDS solution, 5.03 ml deionized water, 75 μl of 10% APS (ammonium persulphate), 7.5 μl TEMED. To prepare a quantity of 15 ml of 4% stacking gel solution the following were added: 1.98 ml 30% acrylamide / Bis Solution 29:1, 3.78 ml 0.5 M Tris HCL (pH 6.8), 150 μl 10% SDS solution, 9 ml deionized water, 75 μl 10% APS, 15 μl TEMED. Electrophoresis was executed at 200 V using a power supply from Bio-Rad power basic. The gels were kept for 1 h in a solution of 40% ethanol and 10% acetic acid for fixation of the protein bands. Gels were stained for 20 h using the QC colloidal Coomassie stain. The gels were destained for 3 h by changing the distilled water three times. Gel DocTM XR+ and ChemidocTM XRS+ Imaging Systems (Bio-Rad Laboratories Inc., Hercules, California, USA) performed gel image acquisition and densitometry. The Image lab software (version 6) operated the instrument. The software was used to determine the protein bands' molecular weights, integrate the peaks, and determine their relative densities.

### Statistical Analysis

A central composite rotatable design (Table 1) and the dependent and independent variables' model relationships (Table 3) were designed using Minitab®19 (USA). The data on the physicochemical, textural, rheological, and proximate composition were analyzed using a one-way analysis of variance (ANOVA) technique. The statistical data were analyzed using the commercial statistical package IBM SPSS (SPSS INC., Chicago, IL, USA). Cheese preparation and analytical measurements were executed in triplicate, and mean values and standard deviations were used in the calculations. Means were related using the least significant difference, and a probability of *p* ≤ 0.05 was considered statistically significant.

**Table 3:** The model equation of independent and dependent variables and its estimated cheeses' estimated constant values.

**Models constants and coefficients**	**Yield (%)**		**Hardness (g)**		**Viscosity (Pa.s)**	
	**Camel cheese**	**Bovine cheese**	**Camel cheese**	**Bovine cheese**	**Camel cheese**	**Bovine cheese**
Constant	11***	11***	729***	1,586***	9,851***	26,374***
C1	−0.005***	−0.037***	+1.44***	−4.36***	+0.01***	−32.93***
C2	−0.06	−0.47*	+17.5**	+12.2*	+123	+433
C3	+0.0002	−0.0006	−0.003**	+0.0037***	−0.006	+0.007
C4	−0.009	+0.027	+0.082	+0.26	−1.69	−11.98*
C5	−0.0006	+0.0007	−0.049	−0.0408	−0.243	−0.63*

^*^Significant terms; p-values: *p < 0.05, **p < 0.01, and ***p < 0.005.

*Response variable value = Constant + C1 × Pressure + C2 × Time + C3 × Pressure^2^ + C4 × Time^2^ + C5 × Pressure × Time + Residuals (all models are significant at p < 0.001)*.

## Results and Discussion

### Milk Composition

The gross composition of camel milk (pH, 6.61; acidity, 0.15%; Lactose, 4.43%; total solids, 12.4%; protein, 2.7%; and fat, 3.1%) was slightly different from that of bovine milk (pH, 6.68; acidity, 0.15%; lactose, 5.08%; total solids, 12.6%; protein, 2.98 %; and fat, 3.4%) in agreement with other researchers (23, 31–33).

### Effect of HPP and Thermal Pasteurization on the Microbial Loads in Camel and Bovine Kinds of Milk

Table 4 revealed that all the pressure-time combinations used in this study were enough to maintain the total plate count and other bacteria below the acceptable limit in camel but not in bovine milk (34). Studies have shown that HPP treatments at 350 and 450 MPa at room temperature and times <15–20 min are not adequate to achieve the reduction of the microbial population (pathogenic and deteriorating) in bovine milk (35). Camel's antimicrobial effects against different pathogens such as *S. aureus, L. monocytogenes*, and *E. coli, Mycobacterium tuberculosis*, and *Salmonella typhimurium* have been reported (36–38). The presence of antimicrobial agents in camel milk may have been the reason for the lower total plate and bacterial counts compared to bovine milk (39). The antiviral and antibacterial properties in camel milk are a result of the presence of peptidoglycan recognition protein (PGRP) enzyme, Igs, N-acetyl-β-glucosaminidase (NAGase), lactoferrin (LF) lactoperoxidase (LP), and lysozyme (LZ,) (40), LF, NAGase, and LZ in camel milk compared to bovine milk (41, 42) while no PGRP is found in cow milk (43).

**Table 4:** Experimental design of the independent variables (pressure, time and at 4°C) on the associated microbial count of camel and bovine HPP and pasteurization milk.

**Sample** **code**	**Pressure (MPa)** **or temperature (C)**	**Time** **(min)**	**Camel milk microbial load (CFU/ml)**				**Bovine milk microbial load (CFU/ml)**			
			**Day 0**		**Day 7**		**Day 0**		**Day 7**	
			**TPC**	**S**	**TPC**	**S**	**TPC**	**S**	**TPC**	**S**
1	550	3	120	50	60	100	NC	10	NC	NC
2	590	6	100	78	22	198	NC	10	NC	NC
3	450	6	170	100	30	150	NC	20	NC	NC
4	450	1	294	100	70	260	NC	50	NC	NC
5	450	6	186	100	56	250	NC	40	NC	NC
6	450	6	200	100	40	100	NC	70	NC	NC
7	450	10	121	200	28	200	NC	50	NC	NC
8	350	9	200	200	100	250	NC	20	NC	NC
9	350	3	290	150	110	200	NC	60	NC	NC
10	450	6	250	150	50	250	NC	70	NC	NC
11	308	6	289	200	76	300	NC	20	NC	NC
12	550	9	112	100	40	100	NC	30	NC	NC
13	450	6	270	100	60	150	NC	40	NC	NC
HTST	75	30 s	10	0	30	50	300	20	NC	NC
LTLT	65	30 min	50	10	43	50	400	50	NC	NC

*TPC, Total plate count; S, Staphylococcus aureus; NC, Not countable, >500 CFU/mL; HTST, High temperature short time; LTLT, low temperature long time. Coliforms, E. coli, Listeria monocytogenes were not detected in day 1 or 7. The impression of the microbial counts did not exceed 15%*.

### The Effects of High-Pressure Processing (HPP) on the Yield, Hardness, and Viscosity of Camel and Bovine Milk Cheeses

**Figure 1:**
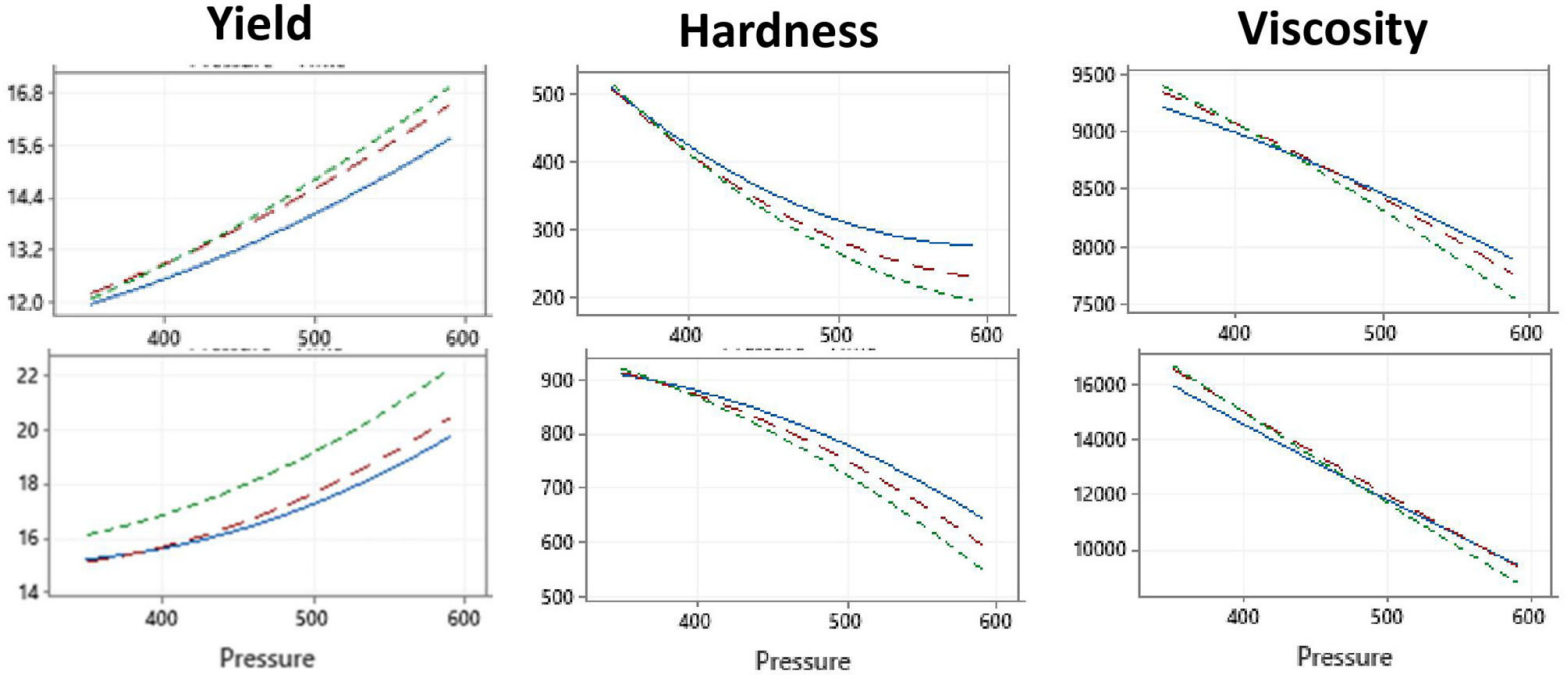
Effect of HPP pressure and time at 4°C on the yield, hardness, and viscosity of camel (Upper panel) and bovine (lower panel) cheeses. Treatment time: 1 min (blue lines), 5 min (red lines), and 10 min (green lines). The illustration shows the effect of pressure—on the yield, hardness, and viscosity at three different times at (1, 5, and 10 min).

Table 1 presents the experimental design for the first study of the effect of high pressure and time (independent variables) on cheese yield, hardness, and viscosity (associated response variables). Plots showing the interaction effects of the independent variables are shown in Table 1. The results clearly show that the increase in pressure results in higher yield but lower hardness and viscosity in both camel and bovine cheeses (Figure 1), which agrees with others (15, 44). The negative correlation between cheese yield and hardness is consistent with our previous observations explained by increased moisture content in the soft cheeses (3). Certain HPP and pressurization conditions may promote extensive whey protein denaturation and interaction with the κ-casein on the surface of the casein micelle (14, 45). Denatured whey proteins were suggested to protect the casein micelles from dissociation and to serve as barriers against their aggregation resulting in cheeses with a relatively open structure and high moisture retention (46). In the case of cheeses made from LTLT and HPP at 350 MPa, this effect might have been minimal explaining the harder texture and lower moisture content.

Table 3 presents the mathematical models that show the significance of the independent variables and their interactions in affecting the camel and bovine cheeses' yield, hardness, and viscosity. The most important terms for both camel and bovine cheeses include the constant, which signifies the inherent differences between the camel and bovine milk, the pressure, and the time and pressure square interactions. The effect of the independent variables on hardness and viscosity were qualitatively similar between the camel and bovine cheeses despite the notable quantitative differences where camel cheeses are significantly (*p* < 0.001) softer than the corresponding bovine cheeses. These observed difference between bovine and camel cheeses is reported to be mainly due to difference in caseins composition. There is a major difference between camel milk and bovine milk caseins. Camel milk caseins consist of α-s1, (22.0%), α-s2, (9.5%), β, (65.0%), and κ (3.5%) whereas bovine milk caseins consist of high percentage of α-casein (38%) followed by 36–39% β-casein and 13% κ-casein (47). Milk κ-casein is the major player in cheese quality because coagulation is initiated when the enzyme chymosin cleavages κ-casein to para-kappa-casein and caseinomacropeptide. Since camel milk has a lower amount of κ-casein due to large casein micelles size. Camel milk coagulation takes a longer time compared to bovine, resulting in weak cheese texture with high moisture content (48–50).

### Comparing the Effects of Pasteurization and HPP Treatments on Cheese Yield and Acidity

The second experiment compared the effect of five treatments: heat pasteurization (LTLT, 65°C, 30 min, and HTST, 75°C, 30 s) and HPP (350, 450, and 550 MPa, 5 min at 4°C each) on the cheese yield, physicochemical, rheological and microstructural properties of camel and bovine milk cheeses. The information presented in Table 2 shows that curd yield HTST treatments resulted was significantly (21 ± 0.2%) and (17 ± 0.3%) higher in both bovine and camel cheeses than all other treatments, i.e., LTLT, and HHP (350, 450, and 550 MPa for 5 min at 4°C) followed by the HHP treatment at 550 MPa (*p* < 0.05). The high yield from HTST-treated milk samples can be due to whey protein denaturation and its interactions with the κ-casein on the surface of the casein micelles (51, 52). High pressurization promotes whey protein denaturation especially β-lactoglobulin which interacts with casein micelle (14, 45). Thus, the denatured whey proteins would serve as barriers against the re-formation of casein aggregates during curd formation, resulting in cheeses with open structure and high moisture content, consequently higher yield (45). Thus, the slight increase in cheese yield is due to the HPP-induced whey proteins' denaturation causing an increase in moisture and fat retention (16, 44, 53). According to our results, HTST-treated camel milk has the lowest suitability for cheese production due to its soft weak curd firmness.

The HPP treatment decreased the titrable acidity and increased the pH of the camel and bovine cheeses significantly (*p* < 0.05). This has been explained by the disaggregation of the colloidal casein micelles and the increased dissolution of ionic calcium phosphate in response to the pressure effect on bovine milk (15, 54–56). Cheese produced from the HTST-pasteurized camel milk samples had the lowest pH and the highest acidity compared to bovine milk samples (*p* < 0.05), which can be explained by enhanced hydrophobic contacts within the casein micelles conferring stability against dissociation with increased temperature (57).

### Comparing the Effects of Pasteurization and HPP Treatments on Cheese Hardness, Rheology, and Microstructure

Bovine milk cheeses had significantly higher textural properties than camel milk cheeses except for cohesiveness (Table 2), which can be explained by the higher content of β-casein with a sticky hydrophobic C-terminal in camel milk (47). The LTLT-treated bovine milk cheese showed improved hardness, gumminess, and chewiness. This can be associated with the milk's faster coagulation, enhancing water drainage, and increasing curd firmness (58). On the other hand, HPP treatment at 350 MPa produced the hardest camel milk, possibly due to the “optimal” disruption of the casein micelles. It was reported that a mild HPP treatment would not cause complete disruption of the casein micelles but rather dissociate parts of their surfaces (59). The micelle fragments would surround fat globules rather than intact casein micelles and make them behave as casein micelles rather than embedded fat globules observed on average in higher pressures (60). Such structures could enhance gel firmness and aggregation by increasing particle associations. The significantly lower textural profile of the bovine cheeses made from HPP-treated milk at 450 and 550 MPa (*p* < 0.05) compared to HPP 350 MPa agrees with previous reports (61). The reduction in firmness upon high-pressure treatments was attributed to increased water retention due to the protein network's hydration. Water in the protein matrix plays a plasticizer role decreasing its elasticity and making it prone to fracture during compression.

Rheology describes the gel system's stress-strain characteristic parameters. G′, the “storage modulus” describes the protein network's elastic (solid) component predicting gel strength (62). The rheological properties (G′, G″, and Viscosity) of bovine milk cheese samples were significantly (*p* < 0.05) higher than that of camel milk cheese samples (Table 2). This could be due to the rapid coagulation of bovine caseins into dense and more interwoven structures (3, 63) compared to soft gel texture in camel caseins (64) as well as yogurts (4, 5). We also observed a decrease in the gel strength and associated rheological properties of cheeses on HPP-treated milk from 350 to 550 MPa (Tables 1, 2; Figure 1). The LTLT samples of bovine milk cheese showed the highest G′, significantly different from the other treatments (*P* < 0.05). In contrast, the viscosity and G′ of HTST-treated milk samples were considerably lower than all the other treatments showing positive relationships with hardness and negative relations with moisture content and yield.

**Figure 2:**
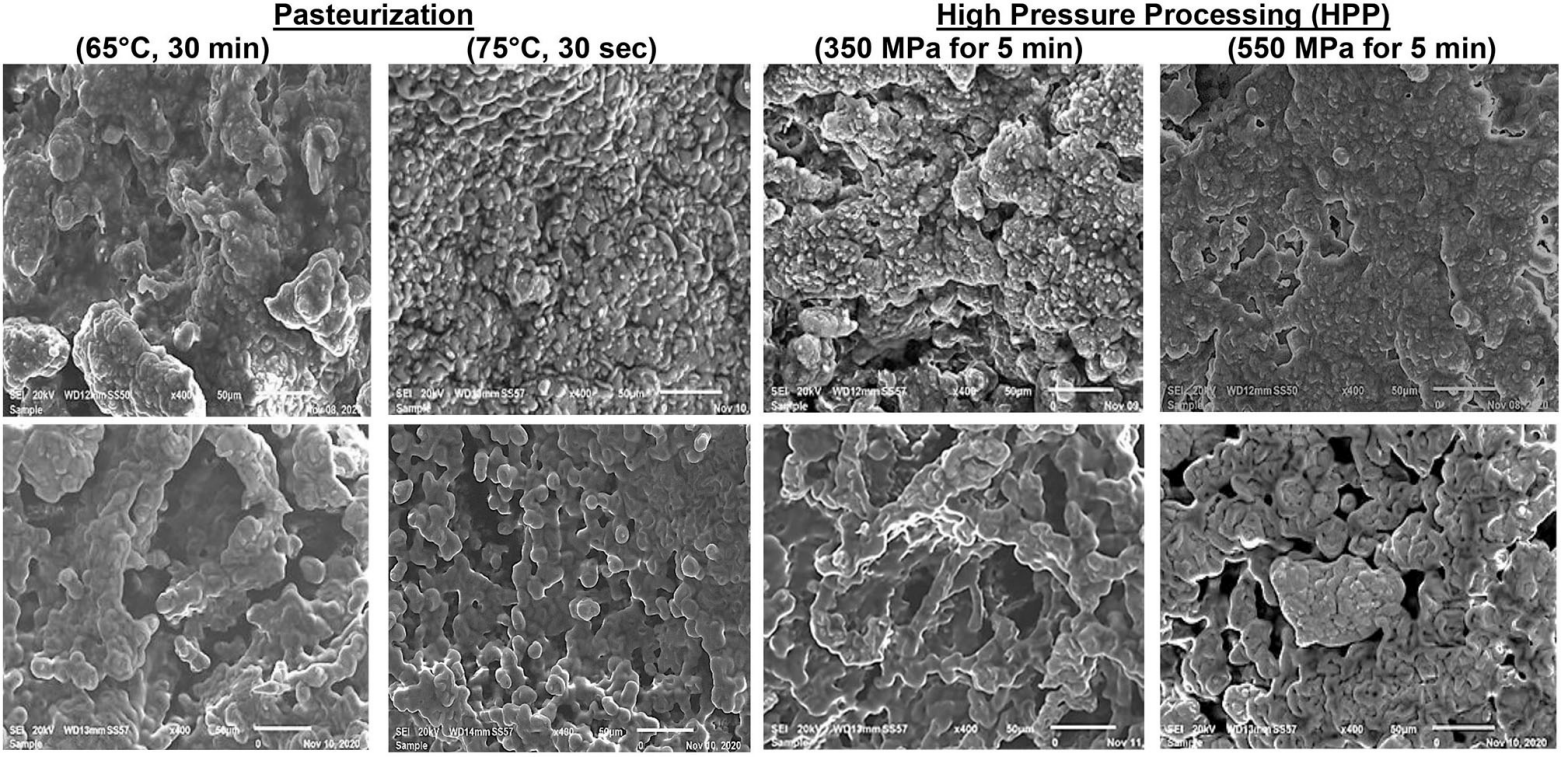
Scanning electron micrograph of camel cheeses (Upper panel) and bovine cheeses (Lower panel) with two pasteurization temperatures and two high-pressure treatments applied to the milk (Magnification: ×400).

Figure 2 presents the microstructures of the two pasteurization levels (LTLT and HTST) and two HHP levels of milk treatment (350 and 550 MPa). Large, irregular lumps characterized the camel and bovine cheeses' microstructure resulting from the LTLT and HHP 350 MPa-treated kinds of milk with granular structures, which permit faster drainage of the whey and enhance cheese hardness (3, 65). On the other hand, cheeses produced from HTST and HHP 500 MPa-treated milk showed tight aggregate strands, homogeneous structures, and continuous networks as observed before (62, 66). The water-holding capacity of curds is directly linked to the gels' porosity (67). Thus, microstructures with smoother protein networks have fewer pore spaces and retain moisture explaining the increased yield and softness (68).

### Proteolytic Activities May Be Involved in the Softness of Camel Milk Cheese

**Figure 3:**
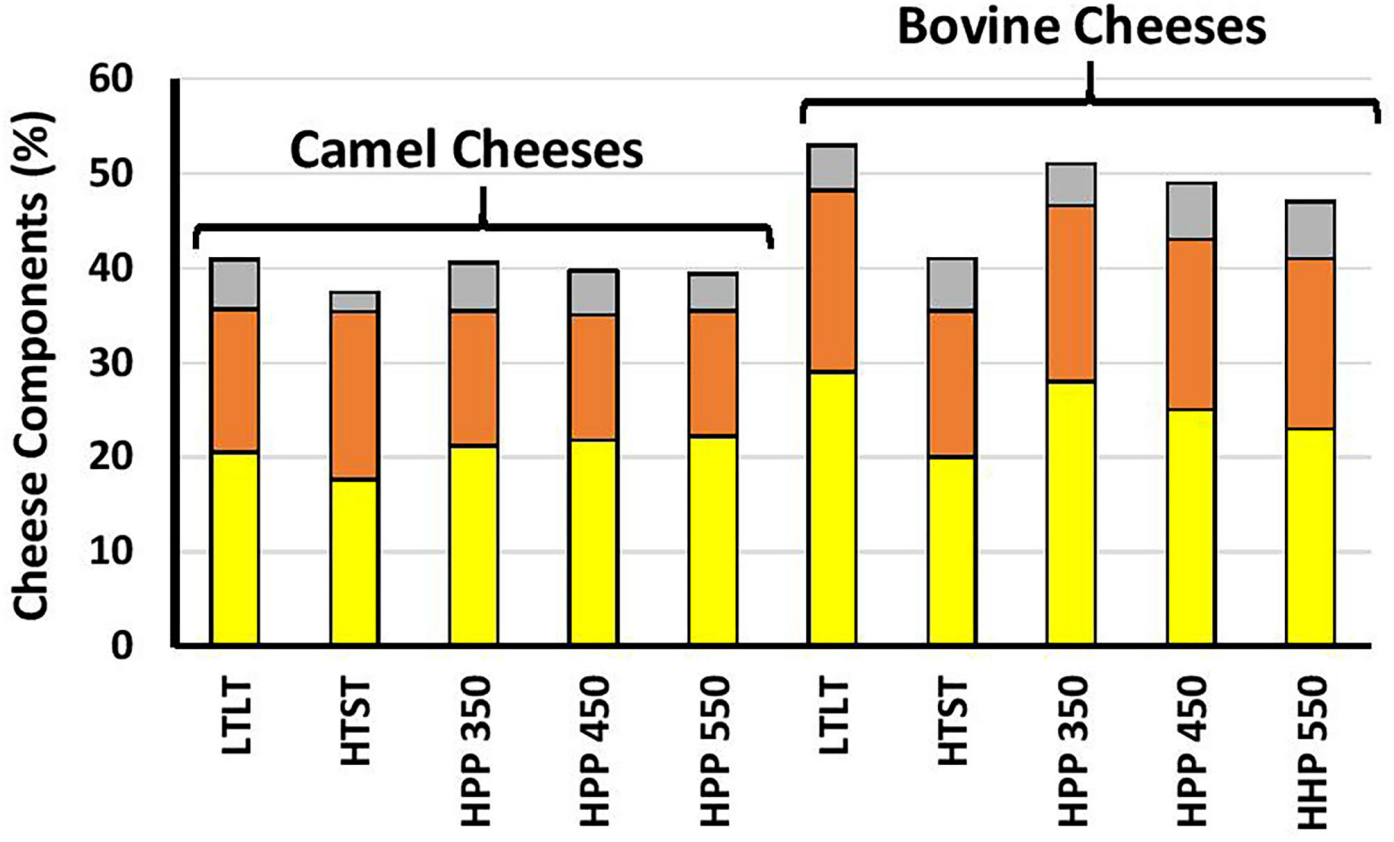
The percentages of fat (yellow), protein (orange), and other solids.

**Figure 4:**
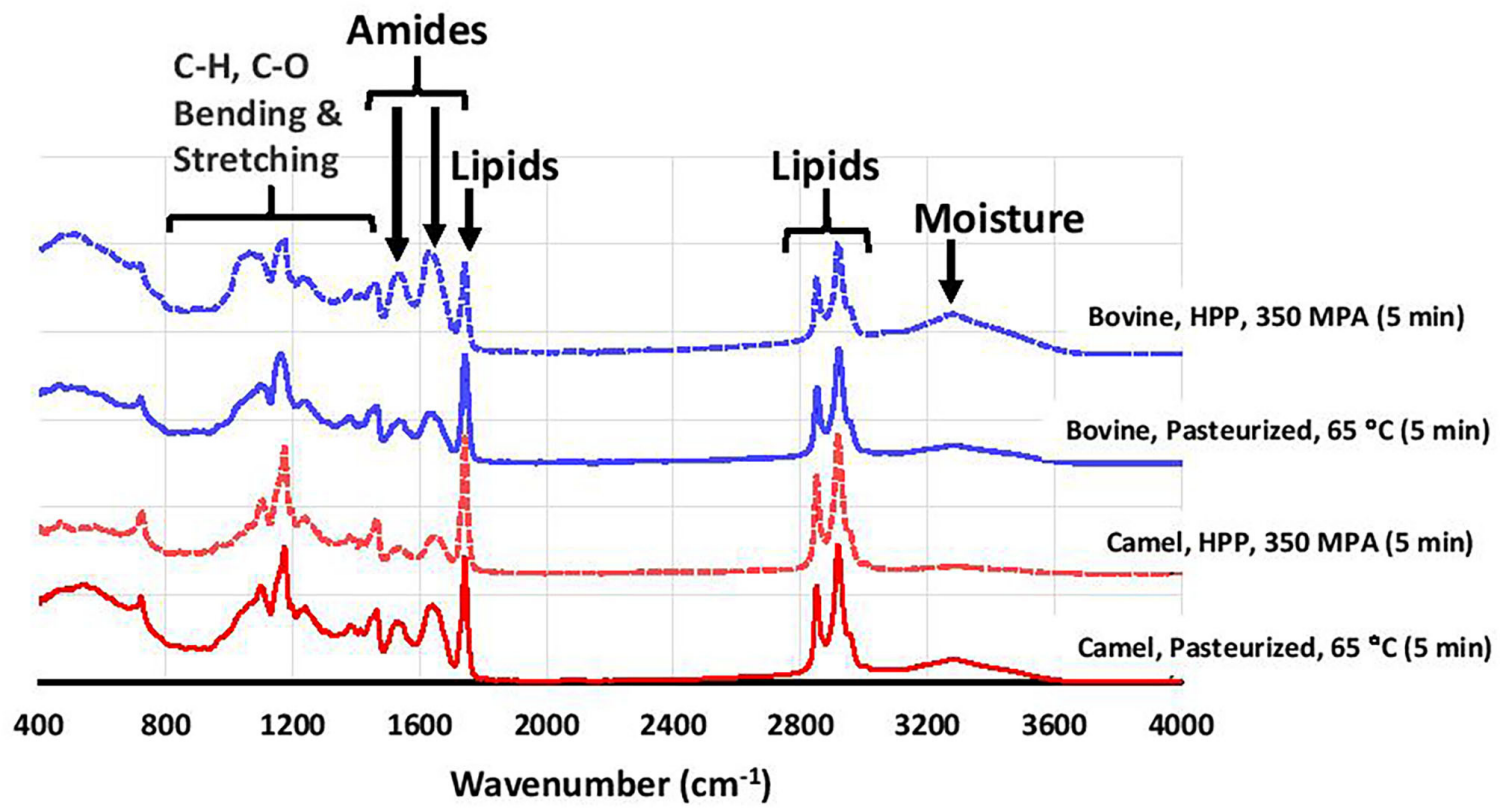
FTIR spectra of selected bovine and camel milk cheeses prepared from heat pasteurization and HHP treatment.

Figure 3 shows that except for the HTST cheese, the fat, protein, and total solids contents were significantly higher in bovine milk cheeses than camel milk cheeses (*p* < 0.001), which is in agreement with previous findings (69, 70). This can be related, at least partly, to the higher level of κ-casein in bovine milk (47). κ-Casein is known to enhance the coagulation properties by forming a denser casein matrix, which reduces the loss of fat and protein to the whey (27, 71). Fourier transforms infrared (FTIR) spectroscopy is a rapid but straightforward technique that analyzes food components based on different functional groups. The FTIR spectra of the cheeses in this study showed typical cheese behavior (Figure 4).

**Figure 5:**
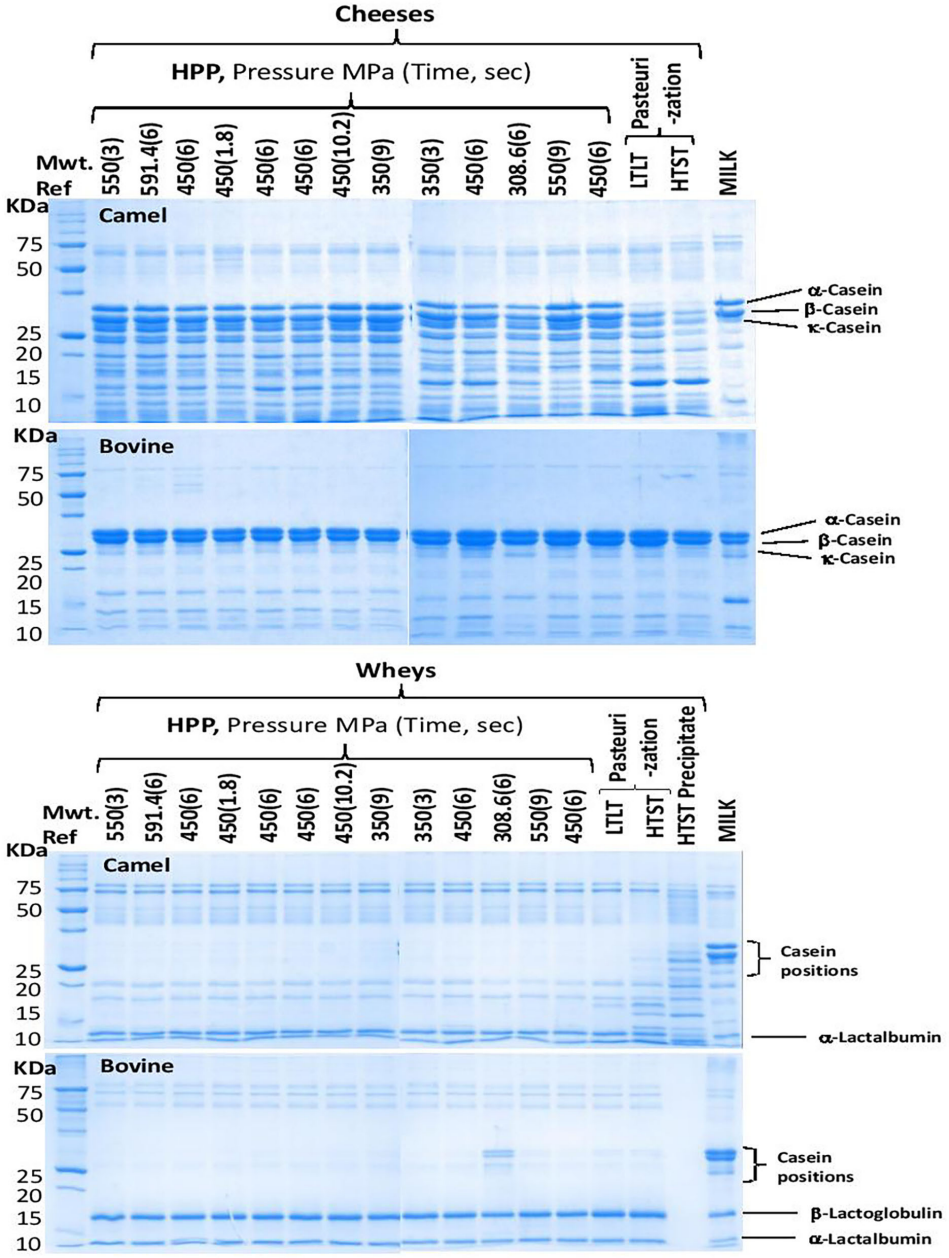
SDS-PAGE electropherograms of the camel and bovine cheeses and whey. For processing conditions (see Table 1).

Figure 5 presents sodium dodecyl polyacrylamide gel (SDS-PAGE) electropherograms of camel and bovine cheeses and wheys. It is observed that the camel milk cheeses show more bands below and above the caseins suggesting extensive proteolysis compared with bovine cheeses. The observed proteolysis may result from the action of two proteolytic enzymes; the residual chymosin used in milk coagulation or the indigenous milk proteinase, plasmin (EC 3.4.21.7) (72–76). The recombinant camel chymosin used in this study is known to hydrolyze bovine and camel κ-caseins at different positions, Phe105-Met106, and Phe97-Ile98, respectively, leading to the release of different macro peptides (77) and possibly fewer hydrolysis products in camel milk because κ-casein is present at very low concentration (3.5%) compared to bovine milk (about 13%) (47). It reported that cheese coagulation by chymosin is slower and weaker in camel compared to bovine milk (3) but the mechanisms behind these differences are still not known. In bovine milk, chymosin hydrolysis of κ-casein is the most important proteolytic reaction during cheese making (78). Of the rennet used in bovine milk coagulation, about 6–10% is retained in the bovine cheese curd (79) but the residues chymosin retained in camel cheese curd to further hydrolyze other caseins requires further investigation.

Notably, the raw and processed camel kinds of milk showed similar proteolytic bands, of less prevalence than those observed in cheese, which are absent in bovine kinds of milk (Figure 5), suggesting that some endogenous proteolytic enzymes in the camel milk may have been activated during cheese processing. Thus, the hydrolysis of camel's milk caseins, predominantly β- and αs1-casein, by the enzymatic plasminogen/plasmin system may contribute to the observed extensive proteolytic activity in camel milk cheeses (80). The numerous peptide bands observed in camel milk cheeses may be explained by plasmin (EC 3.4.21.7) degradation of β-casein, which accounts for 65% of camel milk vs. 40% of bovine milk caseins, and the lack of β-lactoglobulin in this milk (47). γ2-Casein, a C-terminal peptide originating from highly specific proteolysis of β-casein by plasmin, was found in raw camel milk (81). Plasmin activity in milk is affected by the level of its precursor, plasminogen, and some activators/deactivators (73). For example, β-lactoglobulin, the major whey protein in bovine milk that is lacking in camel milk (41, 82), was reported to act as an inhibitor through thiol-disulfide exchange with plasmin causing reduced plasmin activity (83). HPP treatment of bovine milk at pressures higher than 100 MPa was reported to induce β-lactoglobulin denaturation (14).

There are conflicting reports on the effect of HPP on plasmin/plasminogen activity in bovine milk. For example, one study reported that HPP treatment enhances this activity (84) while other studies reported that plasmin activity was not affected by HPP up to 400 MPa for 30 min (14) or 600 MPa for 20 min (85). Thus, the plasmin activity may explain the softness of camel cheeses but it does not explain the hardening effect of HPP on camel milk cheeses. Future studies should investigate the differences between the plasmin/plasminogen systems in camel and their effects on cheese making. As already discussed, the major difference between camel and bovine milk relates to the composition, i.e., the relative percentages of the four caseins, and the nature of the casein micelles in the two kinds of milk. The hydrophilicity/hydrophobicity of the micelle and the access of the hydrolytic enzymes to the reactive sites on the caseins affects the proteolytic activities. In addition, the higher hydration level and concentrations of minerals, mainly calcium, magnesium, phosphate, and citrate, in the casein micelles of camel compared to bovine milk (86) may also play an important role in the micelle structure and its vulnerability to proteolytic attacks (87).

**Figure 6:**
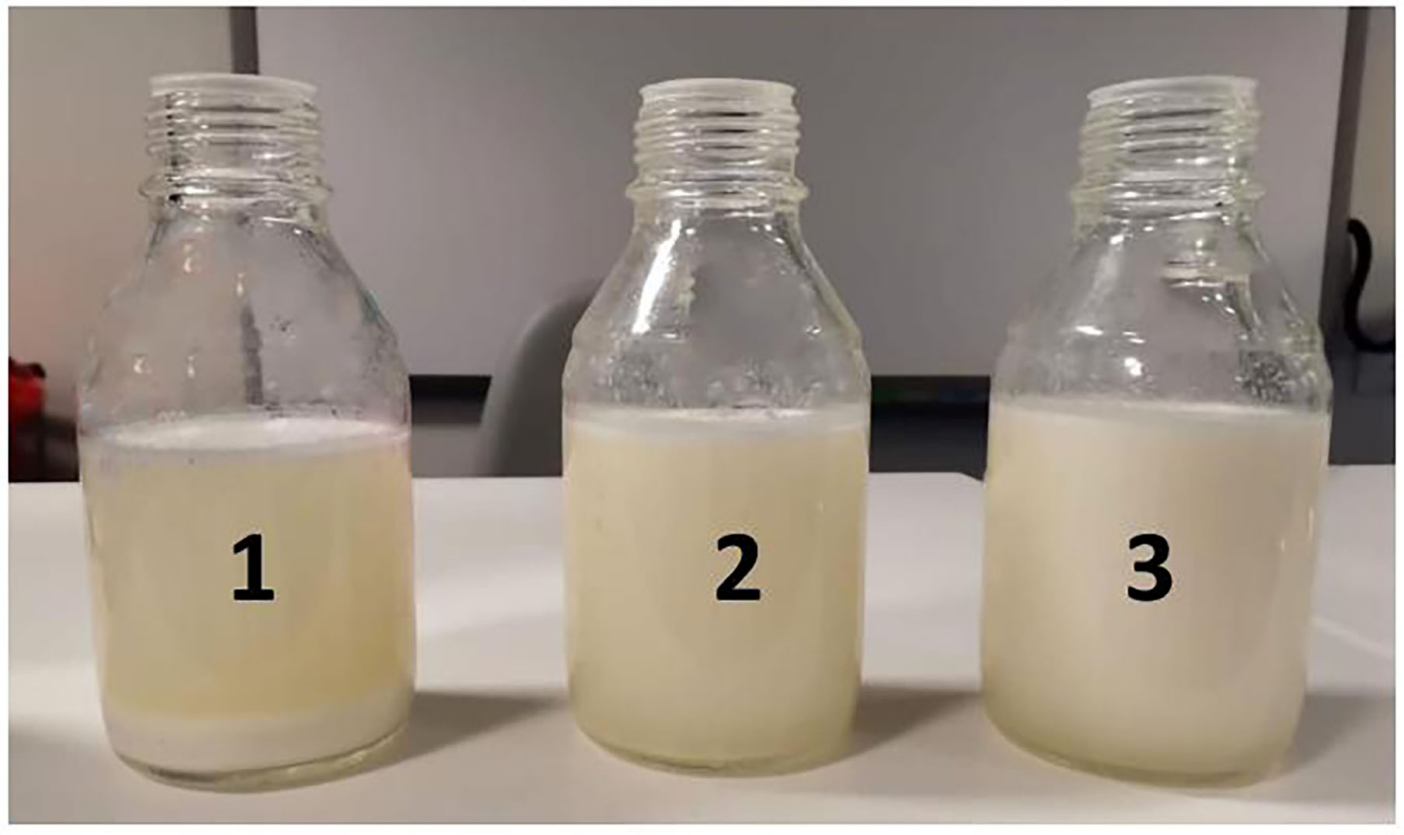
The whey fractions from cheeses produced from camel milk (1) pasteurized (75°C, 30 s), (2) HPP (350 MPa, 5 min at 4°C), and (3) HPP (550 MPa, 5 min at 4°C).

The loose casein micelle structure in camel cheeses may be responsible for water retention and associated with higher yield and lower hardness, viscosity, and rheology. Thus, resulting in loss of more total solid protein and fat into the whey. Our study has revealed camel milk whey had significant (*p* < 0.05) higher total solid protein and fat. LTLT treatment whey had a total solid of (6.9 ± 0.051), fat (1.2 ± 0.00), and protein (1.6 ± 0.04). The results obtained from this study are less than what was reported by (24). While HTST treatment whey had significantly (*p* < 0.05) the highest protein and fat (Table 5). This is due to the infiltration of fat and protein into the whey fraction due to hydrolysis of micro casein during coagulation (Figure 3). The whey fractions from camel milk had tiny casein particles, especially from the HTST-treated milk, where a higher amount of total solid can be observed (Figure 6).

**Table 5:** Chemical of camel and bovine milk whey proteins (*n* = 3).

**Treatment**	**pH**	**Total solids (%)**	**Fat (%)**	**Protein (%)**
**Camel milk whey**				
LTLT, 65°C (30 min)	4.5 ± 0.01^a^	6.9 ± 0.051^c^	1.2 ± 0.00^d^	1.6 ± 0.04^b^
HTST, 75°C (30 s)	4.0 ± 0.01^d^	7.9 ± 0.06^a^	2.0 ± 0.01^a^	1.7 ± 0.02^a^
350 MPa (5 min) at 4°C	4.3 ± 0.02^c^	7.5 ± 0.087^b^	1.3 ± 0.01^d^	1.5 ± 0.02^c^
450 MPa (5 min) at 4°C	4.4 ± 0.01^b^	7.6 ± 0.07^b^	1.4 ± 0.00^b^	1.5 ± 0.02^c^
550 MPa (5 min) at 4°C	4.4 ± 0.02^b^	7.7 ± 0.09^b^	1.4 ± 0.007^b^	1.5 ± 0.03^c^
**Bovine milk whey**				
LTLT, 65°C (30 min)	4.6 ± 0.02^a^	6.0 ± 0.17^d^	1.3 ± 0.02^b^	1.3 ± 0.03^d^
HTST, 75°C (30 s)	4.4 ± 0.03^b^	6.3 ± 0.03^e^	1.4 ± 0.01^b^	1.4 ± 0.01^c^
350 MPa (5 min) at 4°C	4.4 ± 0.02^b^	6.1 ± 0.16^f^	1.3 ± 0.02^c^	1.3 ± 0.02^d^
450 MPa (5 min) at 4°C	4.3 ± 0.02^c^	6.1 ± 0.13^f^	1.3 ± 0.03^c^	1.3 ± 0.02^d^
550 MPa (5 min) at 4°C	4.4 ± 0.01^b^	6.1 ± 0.02^f^	1.3 ± 0.02^c^	1.3 ± 0.02^d^

*A comparison was made between the different treatments for each whey. Values within each column carrying different superscript are statistically different (p < 0.05, n = 3 per treatment)*.

The increase in cheese hardness by HPP may also be affected by factors other than plasmin activity, e.g., disruption and destabilization of the camel milk micelles and enhancement of coagulation. It was reported that when milk is pressurized at room temperature, micelle disruption might enhance the susceptibility of casein to proteolysis by increasing the protein surface area available to the plasmin enzymes as well as the exposure of new substrate sites (80).

## Conclusions

This study investigated the effects of high-pressure milk processing and pasteurization on the yield and physicochemical properties of soft unripe cheeses produced from camel milk as compared with bovine milk. It was found that camel milk cheeses were affected differently from bovine milk cheeses by the different treatments. Camel milk cheeses were relatively softer than bovine cheeses, possibly due to an active endogenous protease proposed to be the plasmin/plasminogen system. The results revealed that mild processing conditions [e.g., LTLT pasteurization (65°C, 30 min) and HPP-treatment (350 MPa, 5 min at 4°C)] were effective in productizing semi-hard cheeses from camel milk. It was also shown that HPP treatment could replace pasteurization HTST (75°C 30 s) in camel milk microbial preservation before cheese production. Further studies are needed to further investigate the possibility of camel cheese production using HPP processing and to evaluate the safety and sensory quality of ripened cheeses. Further studies are also required to identify the proteolytic products in camel milk cheeses, their protein source (s), and their role in camel milk coagulation and cheese quality.

## Data Availability Statement

The original contributions presented in the study are included in the article/supplementary material, further inquiries can be directed to the corresponding author/s.

## Author Contributions

AK-E and RK were in charge of conceptualizing ideas, funding, and supervision the study. MM, HM, AA, and TR performed the laboratory experiments. FH evaluated the SEM results. MM wrote the first draft of the manuscript. All authors reviewed and contributed to the final draft.

## Conflict of Interest

AA and RK were employed by Agthia Public Joint Stock Company (PJSC) Group of Companies. The remaining authors declare that the research was conducted in the absence of any commercial or financial relationships that could be construed as a potential conflict of interest.
